# Role of Genetic Variation in Cytochromes P450 in Breast Cancer Prognosis and Therapy Response

**DOI:** 10.3390/ijms22062826

**Published:** 2021-03-10

**Authors:** Viktor Hlaváč, Radka Václavíková, Veronika Brynychová, Pavel Ostašov, Renata Koževnikovová, Katerina Kopečková, David Vrána, Jiří Gatěk, Pavel Souček

**Affiliations:** 1 Toxicogenomics Unit, National Institute of Public Health, 100 00 Prague, Czech Republic; rvaclavikova@seznam.cz (R.V.); veronikabrynychova@seznam.cz (V.B.); pavel.soucek@szu.cz (P.S.); 2 Biomedical Center, Faculty of Medicine in Pilsen, Charles University, 301 00 Pilsen, Czech Republic; pavel.ostasov@lfp.cuni.cz; 3 Department of Oncosurgery, MEDICON, 140 00 Prague, Czech Republic; renata.kozevnikovova@onko-centrum.cz; 4 Department of Oncology, Second Faculty of Medicine, Charles University and Motol University Hospital, 150 00 Prague, Czech Republic; katerina.kopeckova@fnmotol.cz; 5 Comprehensive Cancer Center Novy Jicin, Hospital Novy Jicin, 741 01 Novy Jicin, Czech Republic; davvrana@gmail.com; 6 Department of Surgery, EUC Hospital Zlin and Tomas Bata University in Zlin, 763 02 Zlin, Czech Republic; gatekj@gmail.com

**Keywords:** breast cancer, cytochrome P450, therapy, response, survival, prognosis, next-generation sequencing

## Abstract

Breast cancer is the most frequent cancer in the female population worldwide. The role of germline genetic variability in cytochromes P450 (CYP) in breast cancer prognosis and individualized therapy awaits detailed elucidation. In the present study, we used the next-generation sequencing to assess associations of germline variants in the coding and regulatory sequences of all human CYP genes with response of the patients to the neoadjuvant cytotoxic chemotherapy and disease-free survival (n = 105). A total of 22 prioritized variants associating with a response or survival in the above evaluation phase were then analyzed by allelic discrimination in the large confirmation set (n = 802). Associations of variants in *CYP1B1*, *CYP4F12*, *CYP4X1*, and *TBXAS1* with the response to the neoadjuvant cytotoxic chemotherapy were replicated by the confirmation phase. However, just association of variant rs17102977 in *CYP4X1* passed the correction for multiple testing and can be considered clinically and statistically validated. Replicated associations for variants in *CYP4X1*, *CYP24A1*, and *CYP26B1* with disease-free survival of all patients or patients stratified to subgroups according to therapy type have not passed a false discovery rate test. Although statistically not confirmed by the present study, the role of CYP genes in breast cancer prognosis should not be ruled out. In conclusion, the present study brings replicated association of variant rs17102977 in *CYP4X1* with the response of patients to the neoadjuvant cytotoxic chemotherapy and warrants further research of genetic variation CYPs in breast cancer.

## 1. Introduction

Breast cancer (Online Mendelian Inheritance in Man, OMIM No. 114480) is the most frequent cancer in females worldwide [1], with 2.1 million new cases diagnosed and more than 620,000 individuals deceased in 2018 [2]. Treatment of so many patients is a serious burden for healthcare systems and calls for an individualized approach. Imbalances in absorption, distribution, metabolism, and excretion of drugs used for cancer therapy influence the drug efficacy and cause adverse drug reactions in some patients. Thus, inter-individual genetic variations in drug-metabolizing enzymes became important in the clinical setting [3]. However, prognostic and predictive biomarkers for precision therapy of breast cancer, e.g., pharmacogenetic variants, are still missing [4].

Cytochromes P450 (CYPs) represent a large superfamily of membrane hemoproteins classified into 18 families in humans [5,6]. CYP monooxygenases oxidize or reduce a broad range of physiological substrates, e.g., sterols and fatty acids [7] and xenobiotics, including drugs [8], and together with ATP-binding transporters, represent the majority of known pharmacogenes [4]. However, of the total number of 57 human CYP coding genes with putative enzymatic activities, only 15 seem to metabolize drugs to date [9], and genetic polymorphisms of *CYP2C9*, *CYP2C19*, and *CYP2D6* belong to the most frequently studied in pharmacogenomics [6]. Although the rest of the CYPs are not involved in drug metabolism perturbations in the homeostasis of steroid hormones, e.g., estrogen may also influence the prognosis and therapy outcome of the patients [10]. Moreover, inhibitors of CYP19 aromatase are frequently used for endocrine therapy of breast cancer patients, while the role of genetic variability of the target gene in the treatment efficacy or adverse effects is yet unexploited for therapeutic decisions [11].

Our pilot pharmacogenomics study followed germline alterations in 509 selected genes and their potential for prognosis and prediction of response to therapy in breast cancer patients [12]. We provided a proof-of-the principle for the study design and established bioinformatics methodology for variant prioritization. Out of all genes whose coding and regulatory sequences were screened by the next-generation sequencing (NGS) approach, only a few variants were validated in the replication phase. We down-sampled the first-phase results by the synthesis of in silico predictions and statistically significant clinical associations. This strict process resulted in considerably smaller numbers of variants for replication, and some potentially useful biomarkers of prognosis or prediction of therapy outcome potentially remained unexplored.

In the present study, we used less-strict criteria for investigating the importance of germline genetic variability in coding, untranslated regions (UTR), and adjacent regions of all human members of the CYP superfamily for prognosis and response to the neoadjuvant cytotoxic therapy (NACT) of breast cancer patients. First, we correlated variants with the response of patients to NACT and disease-free survival (DFS). We then thoroughly reviewed haplotypes and gene dosage and corrected results for multiple testing. This study has not addressed the functional relevance to enable also the identification of purely correlative biomarkers. Prioritized variants underwent confirmation in a large cohort of breast cancer patients from a single population. Taken together, the present study brings a more detailed view of the relevance of germline genetic variability of CYPs for breast cancer prognosis and therapy outcome.

## 2. Results

### 2.1. Evaluation Phase

The clinical characteristics of the patients (n = 105) are in Table S1. A subgroup of patients received NACT (n = 68), and the response to this treatment was available. The rest of the patients received adjuvant cytotoxic therapy based on monotherapy or combinations of anthracyclines, cyclophosphamide, 5-fluorouracil, and taxanes. The mean follow-up of the patients was 70 ± 28 months.

The average coverage was 92.5 ± 32.2 with 94.6% of the captured regions covered at least 10 times. Altogether, we found 1274 variants in exonic and adjacent intronic sequences. The human CYP superfamily (57 genes) contained on average 22.4 ± 13.0 variants per gene. The lowest counts of variants were found in *CYP21A2* (one variant), *CYP26C1* (seven), *CYP3A4*, and *CYP19A1* (both eight variants). On the other hand, *CYP4V2* (58 variants), *CYP4F8* (56), and *CYP4F12* (55) were the most polymorphic genes. Of the total number of 1274 variants, 302 were in exons, 685 intronic, and 210 were in 3′ or 5′ UTRs according to the National Center for Biotechnology Information (NCBI) Reference Sequence Database (RefSeq; https://www.ncbi.nlm.nih.gov/refseq/) (Table 1).

On average, each patient showed 336 ± 27 variants. We found 14 loss-of-function (LOF) truncating variants that were either affecting the stop codon (stop-gain) or frameshift insertions or deletions (indels). Out of exonic variants, 178 were non-synonymous single-nucleotide variants (SNVs) and 99 synonymous SNVs (Table 2). In total, 568 variants (45%) had minor allele frequency (MAF) > 0.05; the rest, 706 variants, had MAF 0.05 or below.

**Figure 1:**
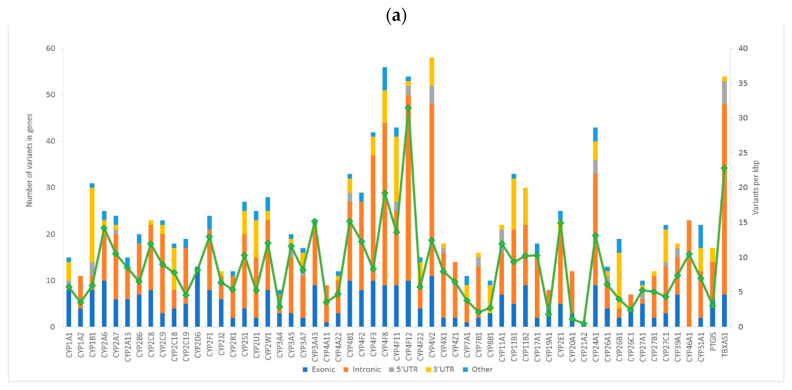

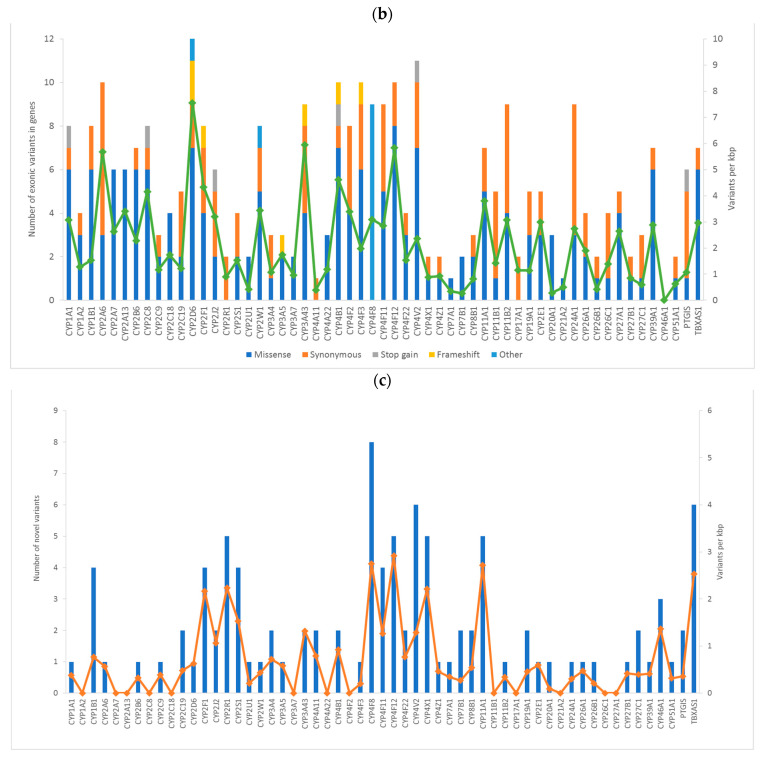
Distribution of alterations in individual cytochromes P450 (CYPs). The picture shows: (**a**) the frequency of genetic alterations according to their functional classes; (**b**) The frequency of genetic alterations according to their exonic functional classes analyzed by the National Center for Biotechnology Information Reference Sequence Database (https://www.ncbi.nlm.nih.gov/refseq/) (Access on: 29 September 2019); (**c**) The distribution of novel variants. Numbers of variants, normalized to the transcript length in kilobase pairs (kbp), are depicted for each gene by the overlaid line in each plot on the right axis.

Altogether, 103 (8%) of the variants were novel (i.e., not found in dbSNP Build 150). Out of these, ten had MAF > 0.01 and were classified as single-nucleotide polymorphisms (SNPs) in the Czech population. The distribution of variants according to their functional classes and frequencies of novel variants in gene groups is shown in Figure 1.

Variants departing from Hardy–Weinberg equilibrium (*p* < 0.01, n = 76) were excluded from analysis. Further, we selected variants with MAF > 0.05 to achieve adequate statistical power in the confirmation phase. Besides, variants with the missing data in more than 50% of patients were excluded (n = 25). This filtration process resulted in a set of 564 variants which were further evaluated for associations with the response of patients to the NACT and DFS. We found 32 variants associated with the response to the NACT and other 32 variants with DFS. Following haplotype evaluations, 27 variants were considered tagging other selected variants (r^2^ > 0.8) and not assessed further. The gene dosage relationship was then evaluated for variants associated with DFS, and variants not fulfilling this condition were excluded (n = 13). Neither of these variants was significant in the recessive genetic model (variant allele versus common homozygote). Following these control steps, we prioritized 24 variants (23 SNVs and 1 insertion, Table S2) for the confirmation phase in a larger cohort of breast cancer patients, but optimization of three variants failed during the reaction design. One variant was then included in the list (rs593421) based on haplotype tagging (r^2^ = 1) to replace the variant rs79882219, whose analysis failed. No tagging variants (r^2^ > 0.8) were available to replace the rest of failed variants.

### 2.2. Confirmation Phase

The clinical characteristics of the patients (n = 802) are shown in Table S3. A subgroup of patients treated with the NACT comprised 168 patients. In total, 371 patients received adjuvant cytotoxic therapy. Patients with localized disease and the generally good prognosis did not receive any further treatment (n = 83), and a portion of patients was treated only with hormonal therapy (n = 311). The mean follow-up of the patients was 76 ± 30 months.

All successfully genotyped variants (n = 22) were in Hardy–Weinberg equilibrium. Less than 1% of theoretical data points were missing due to the uncertainty in genotype calling or absent signal. Table 3 summarizes genotypes’ distribution of the variants in the confirmation phase. The MAFs of assessed variants in the confirmation set were comparable to MAFs observed in the evaluation set.

**Figure 2:**
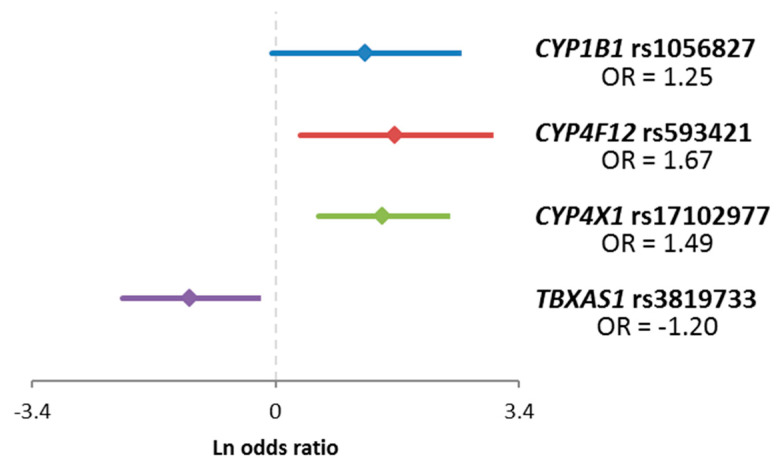
Common odds ratios of variants significantly associated with the response to neoadjuvant chemotherapy. OR = odds ratio.

**Figure 3:**
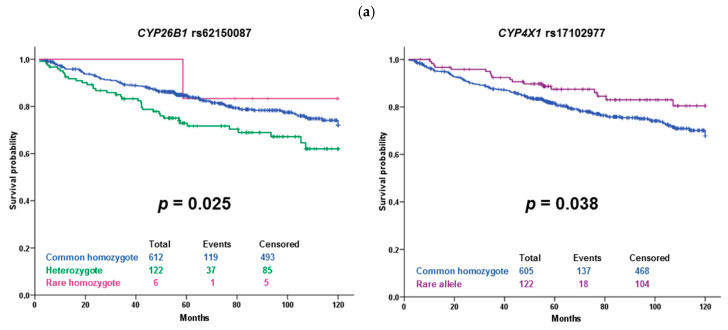

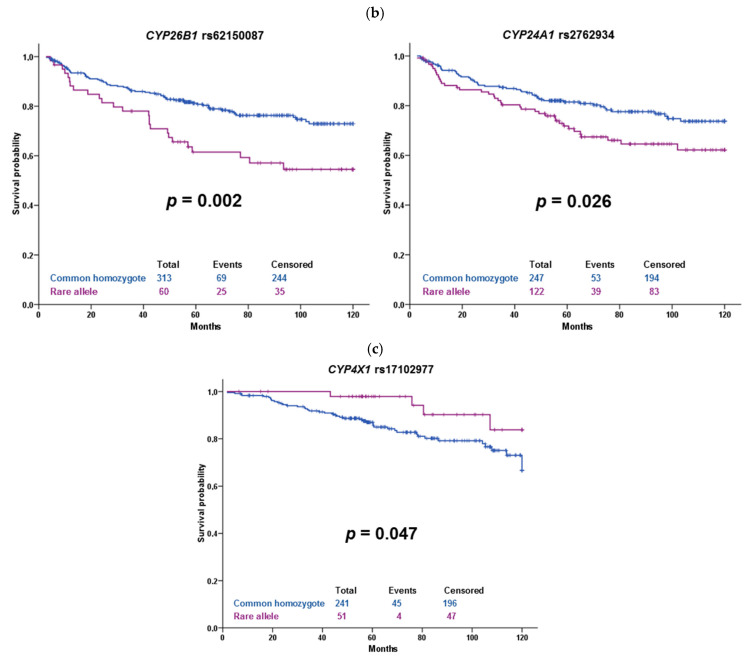
Kaplan–Meier plots with significant associations of cytochrome P450 variants with disease-free survival. (**a**) All patients, (**b**) subgroup of patients treated with the cytotoxic therapy, and (**c**) subgroup of patients treated only with the hormonal therapy. Blue line represents the common homozygous genotype, green heterozygote, and magenta rare homozygote. Violet color represents rare allele carriers (recessive model). Significance was evaluated by the log-rank test; numbers show individuals in the compared groups.

For validation purposes, we also evaluated associations of variants with the response to the NACT and DFS of patients in the confirmation set. For SNPs with less frequent homozygous genotypes (n < 5), the recessive genetic model was used to maintain sufficient statistical power. Significant results for response are in Table 4 and Figure 2. Subsequently, we evaluated associations of variants with DFS of all patients and patients stratified according to the received therapy. Significant results for all patients with complete follow-up data (n = 744) are in Figure 3a, for patients treated with cytotoxic therapy (n = 373) in Figure 3b, and for patients treated only with hormonal therapy (n = 312) in Figure 3c.

Association of SNP rs17102977 in *CYP4X1* with the response to the NACT passed the correction for multiple testing and therefore can be considered clinically and statistically validated. The rest of the associations with response to NACT did not pass the false discovery rate (FDR) test (Table 4). Several significant associations with DFS were observed as well, but none of these associations remained significant after the adjustment for multiple comparisons. Interestingly, the variant rs17102977 in *CYP4X1*, associating with the response, also associated with DFS in the group of all patients without regard to therapy and in the subgroup treated only with hormonal therapy (Figure 3a,c).

In a multivariate analysis adjusted to tumor size and grade, presence of regional lymph node metastasis, and estrogen receptor status, the rs17102977 in *CYP4X1* significantly associated with DFS in the unselected set of patients (*p* = 0.048; hazard ratio, HR = 1.69; 95% confidence interval, CI = 1.01–2.85). The rs62150087 in *CYP26B1* significantly associated with DFS in a group of cytotoxic therapy-treated patients (*p* = 0.016, HR = 0.54, CI = 0.33–0.89), but not in unselected patients (*p* = 0.122). The rs2762934 SNP in *CYP24A1* in a subgroup of the cytotoxic therapy-treated group was insignificant in multivariate analysis (*p* = 0.075).

To clarify the effect of intrinsic molecular subtypes on the prognosis, we stratified patients into four groups: Luminal A, Luminal B, HER2-enriched, and triple-negative breast cancer (TNBC) according to their subtypes. Subsequently, we assessed the associations with DFS for each subtype separately. Results are depicted in Table 5 and Figure S1. Variant rs62150087 in *CYP26B1* was significantly associated with DFS in the HER2-enriched group of the patients regardless of the therapy (all patients or patients who received cytotoxic therapy). Rs2762934 in *CYP24A1* was significantly associated with DFS in a subgroup of TNBC patients treated with cytotoxic therapy. Rs17102977 in *CYP4X1* was insignificant in all subtypes (Table 5).

## 3. Discussion

We analyzed associations of genetic variants in all human CYP monooxygenases with chemotherapy outcome and survival of breast cancer patients. Firstly, we genotyped all coding sequences and surrounding areas using the next-generation sequencing. Variants significantly associated with response to NACT and DFS of the patients using Chi-square and log-rank tests with permutations were further assessed in a large cohort of breast cancer patients (n = 802) by competitive allele-specific PCR. Of the total number of 22 variants selected for validation, the results were confirmed, in a large cohort, for six of them.

In total, we found 1274 variants in a set of 105 breast cancer patients used for the evaluation phase. We found 14 LOF variants. Of these, six were stop-gain mutations, and eight were frameshift indels. However, the MAF of these variants was too low to maintain the statistical power for correlation with clinical data precluding their further study. Thus, we evaluated 22 common variants in a large confirmation cohort of patients and assessed their associations with DFS and response to NACT.

The substitution rs17102977 in *CYP4X1* intron is associated with both response of the patients to the NACT and DFS of hormonally treated patients. It is also prognostic in patients unselected according to the therapy. The association of rs17102977 with the response to NACT was significant after correction to multiple testing and thus can be considered validated in both datasets. Patients carrying the rare allele G were more often poorly responding to chemotherapy than the patients with the wild-type genotype AA. Intriguingly, patients bearing rare alleles had longer DFS than wild-type patients. Although the frequency of the rare allele in the European non-Finnish population in GnomAD is 0.08, we found no record for this variant in the scientific literature (PubMed). The gene *CYP4X1* encodes an orphan CYP enzyme expressed mainly in the brain, aorta, or breast [13,14]. According to recent studies, this enzyme catalyzes epoxidation of endogenous cannabinoid anandamide and arachidonic acid [15,16]. Its important paralog is *CYP4Z1* [17]. The role of *CYP4X1* in cancer has been proposed as well. Protein expression of CYP4X1 was associated with increasing tumor grade in tissue microarrays from 170 breast cancer patients detected by immunohistochemistry [18]. Recently, a lower gene expression of *CYP4X1* was associated with shorter overall survival of Chinese gastric cancer patients treated with capecitabine and oxaliplatin [19]. Two SNPs in *CYP4X1* were also associated with the early onset of Creutzfeldt-Jakob disease in Italian patients [20]. The endocannabinoid system is involved in various physiological processes, including inflammation, immunomodulation, or suppression of different cancers, e.g., breast cancer [21]. Therefore, CYP4X1 might play a role in response to cancer chemotherapy through physiological processes. The role of rs17102977 in cancer is unknown. According to the GTEx portal (https://www.gtexportal.org) (Access on: 10 February 2021), the expression quantitative trait loci (eQTL) analysis showed that rs17102977 was significantly associated with *CYP4A22* gene expression in the brain, but not in the breast tissue. No significant association with *CYP4X1* gene expression was found. Therefore, further elucidation of the function of rs17102977 and mainly the whole gene locus 1p33 containing also *CYP4B1*, *CYP4A11, CYP4Z1*, and *CYP4A22* genes is needed. This locus also contains mitogen-activated protein kinase *MNK1*, frequently studied in cancer research.

The variant rs62150087 (500 bp downstream of *CYP26B1*) was associated with DFS in our sets of patients. Carriers of the rare allele G treated with cytotoxic therapy had significantly shorter DFS than wild-type patients, but the observed *p*-value of 0.002 does not guarantee a true positive association after correction for multiple testing. However, this borderline significant result is interesting. A genome-wide association study (GWAS) on the Chinese population identified *CYP26B1* as a candidate gene for esophageal squamous cell carcinoma risk. According to the authors, a variant rs138478634 in *CYP26B1* influences the risk through catabolism of an anticancer nutrient all-*trans* retinoic acid [22]. Considering the ability of CYP26B1 to metabolize retinoic acid, there is support for the observed effect of rs62150087 on worse patient survival. However, the influence of this downstream variant on enzyme function or expression is not known, and no significant eQTLs were found in any of the available tissues.

Among other associations found, the variant rs1056827 in *CYP1B1* was associated with the response of the patients to NACT. Homozygotes of the variant allele A were more often poorly responding to chemotherapy than wild-type patients or heterozygotes. This SNP 355G>T changing Alanine to Serine in codon 119 is associated with susceptibility to colorectal [23,24], breast cancer [25], and primary open-angle glaucoma [26]. According to ClinVar, this variant is considered benign, and thus no clear explanation of the observed association is available now. Also, the link between breast cancer risk and therapy response is unclear. CYP1B1 oxidizes estrogen as well as a wide variety of xenobiotics [14,27]. This fact perhaps could help to explain associations with both risk and therapy outcome and focus further studies. Similarly, synonymous variant rs593421 in *CYP4F12* has no clear support for an observed association with response. *CYP4F12* was cloned originally from the intestine and liver, its recognized substrates are fatty acids [13,14]. Also, intronic variant rs3819733 in thromboxane synthase (*TBXAS1)* was associated with response to NACT in our patients. In the literature, lower *TBXAS1* expression was associated with higher grade and poor prognosis of breast cancer patients [28]. On the contrary, high expression was associated with worse overall survival of patients with diffuse low-grade glioma [29]. CYP24A1 is responsible for vitamin D metabolism [30]. SNP rs2762934 in an intronic region of *CYP24A1* was associated with an increased risk of breast cancer [31], ischemic stroke [32], and hypertension [33]. We have seen an association of rs2762934 with DFS of patients treated with cytotoxic therapy, especially in the TNBC subgroup. Taken together, these associations are interesting but should be treated with caution because of their low significance level after FDR correction, as well as considering vague support in the literature.

The present study has some limitations. First, the modest size of the evaluation set may be seen as a study limitation, because it precludes assessment of the importance of very rare (MAF < 0.001) and rare (<0.01) variants. In light of the recently acknowledged contribution of rare variants to inter-individual variability in drug response [3], this limitation needs attention in future studies in precision oncology. On the other hand, we consider ethnical homogeneity and robustness of clinical follow-up as notable study benefits. Moreover, we have employed the multivariate analyses adjusted to major disease characteristics and stratified patients according to intrinsic molecular subtypes to circumvent the frequently overlooked issue of non-homogeneity from the clinical point of view. Major conclusions of the study remained unchanged. Up to that, further elucidations are needed to explore additional genetic components, e.g., non-coding sequences, copy numbers and structural variations, somatic mutations, etc., of the CYP superfamily in breast cancer.

## 4. Materials and Methods

### 4.1. Patients

In the evaluation phase of the study, we included 105 breast cancer patients diagnosed in the Medicon in Prague and the Hospital Atlas in Zlin (both in the Czech Republic) in the period 2006–2013. The NACT administered to patients (n = 68) before tumor resection contained regimens composed of 5-fluorouracil, anthracyclines, cyclophosphamide (FAC or FEC), and/or taxanes. The rest of the patients underwent adjuvant postoperative treatment based on the same drug combinations. Table S1 illustrates the clinical data of the patients.

We used 802 breast cancer patients in the confirmation phase. The patients’ recruitment proceeded between 2001 and 2013 in Medicon and the Motol University Hospital (both in Prague) and the Hospital Atlas in Zlin. Patients received neoadjuvant/adjuvant cytotoxic therapy or hormonal therapy (clinical data in Table S3).

We described the recruitment schema before [12,34]. DFS was defined as the time elapsed between surgery and the first disease relapse, including local relapses. The response to the NACT was evaluated using the Response Evaluation Criteria In Solid Tumors (RECIST) based on imaging data retrieved from medical records [35].

Procedures performed in the present study followed the 1964 Helsinki Declaration and its later amendments or comparable ethical standards. The Ethical Commission of the National Institute of Public Health in Prague approved the study protocol (approval code no. 9799-4 (issued on 30 January 2008), 15-25618A (6 August 2014), and 17-28470A (22 June 2016)). All patients were informed about the study, and those who agreed and signed informed consent of the patient further participated in the study.

### 4.2. Panel Sequencing—Evaluation Phase

Blood samples were collected during the diagnostic procedures using tubes with K3EDTA anticoagulant and genomic DNA was isolated from human peripheral blood lymphocytes by the standard phenol/chloroform extraction and ethanol precipitation.

All 57 human CYP genes were sequenced using target enrichment protocol on MiSeq (Illumina, San Diego, CA, USA) platform as described previously [12]. Briefly, reads were mapped on a reference sequence hg19 using Burrows–Wheeler Alignment (BWA) mem tool [36], base and indel recalibration as well as the short indels and SNVs discovery was performed using the genome analysis toolkit (GATK) [37]. Variant annotation was performed with the help of Annovar [38]. For details of the library preparation, target enrichment, data processing, and variant calling, see [12].

### 4.3. Genotyping—Confirmation Phase

Variants in the confirmation phase were commercially genotyped by the allelic discrimination method (KASP™, LGC Genomics, Hoddesdon, UK) using primers and probes designed by the service provider. Of note, 10% of the samples were analyzed in duplicates for quality control. The genotyping concordance between duplicate samples exceeded 99%.

### 4.4. Quantitative Real-Time PCR

Quantitative real-time PCR (qPCR) and evaluation of results were performed as described previously [34]. The relative standard curve was generated from five log dilutions of one non-neoplastic control tissue sample (calibrator). Amplification efficiencies (E) for each reference and target gene were calculated applying the formula E = 10^−1^/slope^−1^. *EIF2B1, MRPL19*, and *IPO8* were used as the most stable reference genes for data normalization [34]. The qPCR study design adhered to the MIQE Guidelines (Minimum Information for Publication of Quantitative Real-Time PCR Experiments [39].

### 4.5. Statistical Analyses

DFS was calculated for groups of patients divided by the common homozygous, heterozygous, and rare homozygous genotype in the evaluation phase. The Kaplan–Meier plot served for visual inspection of gene dosage, and the log-rank test was used for evaluation of statistical significance among survival curves of patients with given genotypes or alleles. We set the study follow-up end to 120 months (10 years), and thus all subjects with DFS exceeding 120 months were censored. The response of patients to the NACT was set to “good” in the case of complete or partial pathological remission (CR/PR) and “poor” for stable or progressive disease (SD/PD). We evaluated associations between genotypes (common homozygous, heterozygous and rare homozygous) and response using the Pearson Chi-square test. The adjusted *p*-value was calculated for each variant and each of these tests. The adjusted *p*-value for the log-rank test was based on 100 permutations of original data. A *p*-value of less than 0.05 after adjustment for multiple testing was considered statistically significant. Variants significantly associating with either DFS or the response to NACT in the evaluation phase entered the confirmation phase of the study.

In the confirmation phase, the Pearson Chi-square test and the log-rank tests were used as described above. For the evaluation of variant effects, recessive, dominant, and additive genetic models were employed. Multivariate analysis was done using the Cox proportional hazards model with tumor size and grade, lymph node metastasis, and estrogen receptor expression as covariates. Associations of variants with transcript levels were assessed by one-way ANOVA. Adjusted *p*-values were calculated using the Benjamini–Hochberg FDR test as a correction for multiple testing [40]. Haplotype analysis was conducted in HaploView 4.2 (Broad Institute, Cambridge, MA, USA). Statistical analyses were performed using R (v3.5) with packages VariantAnnotation (v3.8), tidyverse (v1.2.1), survival (2.43), survminer (v0.4.3) and survMisc (v0.5.5) and the statistical program SPSS v16.0 (SPSS, Chicago, IL, USA).

The sequencing data that support the findings of this study are openly available in Sequence Read Archive (SRA, https://www.ncbi.nlm.nih.gov/sra), under accession No. PRJNA510917.

## 5. Conclusions

In conclusion, we have seen associations of selected variants in CYP genes with the response to NACT and with the DFS of the patients. Apart from rs17102977 found in an intronic region of *CYP4X1*, which was associated with response to NACT, no variants passed the correction for multiple testing. Protein expression of *CYP4X1* was already studied in breast cancer, but the role of its genetic variation is unexplored. Another intriguing association we found, though not FDR-validated, is an association of rs62150087 (*CYP26B1*) with shorter DFS. CYP26B1 metabolizes retinoic acid, a compound with potential anticancer abilities. In this regard, a recent GWAS revealed an association of *CYP26B1* with esophageal squamous cell carcinoma risk. However, no studies describing associations with survival are available to date. Taken together, genetic polymorphisms in CYP genes may play a role in the prediction of breast cancer therapy and perhaps modify the prognosis of the patients. More studies must follow to confirm these associations.

## Figures and Tables

**Table 1:** Observed alterations in cytochromes P450 divided by function according to Annovar.

Function	Total	Percentage
Intronic	685	53.8
Exonic (coding)	302	23.7
3′UTR	167	13.1
5′UTR	43	3.4
Upstream ^1^	45	3.5
Downstream ^1^	19	1.5
Intergenic	10	0.8
Splicing ^2^	3	0.2

Footnotes: ^1^ Variants are 1 kb from transcription end/start site; ^2^ Variants are 2 bp within splice junction; UTR = untranslated region.

**Table 2:** Overview of the observed exonic alterations in cytochromes P450 by coding consequence.

Classification	Total	Percentage
Non-synonymous SNV	178	58.9
Synonymous SNV	99	32.8
Stop-gain	6	2.0
Frameshift deletion	4	1.3
Frameshift insertion	4	1.3
Non-frameshift deletion	2	0.7
Unknown	9	3.0

SNV = single nucleotide variant.

**Table 3:** Distribution of genotypes for variants assessed in the confirmation phase.

Gene	SNP ID ^1^	Genotype Distribution ^2^			Minor Allele Frequency	
		Common Homozygotes	Heterozygotes	Rare Homozygotes	Confirmation Set	Evaluation Set
*CYP1B1*	rs1056827	362	354	77	0.32	0.34
*CYP2S1*	rs184623	308	379	100	0.37	0.38
*CYP2W1*	rs3808348	538	237	23	0.18	0.20
*CYP2W1*	rs12701220	533	239	25	0.18	0.11
*CYP4A11*	rs3890011	459	291	46	0.24	0.27
*CYP4F2*	rs2074900	367	343	83	0.32	0.32
*CYP4F2*	rs3093198	398	325	73	0.30	0.29
*CYP4F8*	rs714772	506	258	35	0.21	0.25
*CYP4F8*	rs4646522	225	401	158	0.46	0.42
*CYP4F12*	rs593421	416	308	54	0.27	0.29
*CYP4F12*	rs593818	230	373	187	0.47	0.43
*CYP4F12*	rs2074568	518	211	23	0.17	0.21
*CYP4V2*	rs62350517	693	104	4	0.07	0.08
*CYP4X1*	rs17102977	653	125	8	0.09	0.10
*CYP24A1*	rs2259735	246	365	155	0.44	0.39
*CYP24A1*	rs2762934	549	231	17	0.17	0.17
*CYP24A1*	rs6022999	496	251	50	0.22	0.21
*CYP24A1*	rs10623012	294	382	105	0.38	0.32
*CYP26B1*	rs61138718	606	183	12	0.13	0.11
*CYP26B1*	rs62150087	661	132	6	0.09	0.07
*CYP27C1*	rs12476709	236	379	174	0.46	0.47
*TBXAS1*	rs3819733	590	195	14	0.14	0.15

^1^ Reference number in dbSNP (https://www.ncbi.nlm.nih.gov/snp/) (Access on: 8 August 2019); ^2^ Genotypes do not sum up to 802 due to missing data; SNP = single nucleotide polymorphism.

**Table 4:** Cytochrome P450 variants significantly associating with the response of patients to the neoadjuvant cytotoxic therapy in the confirmation set.

Gene	SNP ID	Genotype	Good Response ^1^	Poor Response ^1^	χ−Square	*p*
*CYP1B1* ^3^	rs1056827	C allele	122	35	3.96	0.047/0.339 ^2^
		AA	5	5		
*CYP4F12*	rs593421	TT	63	22	8.81	0.012/0.130 ^2^
		TC	57	12		
		CC	4	6		
*CYP4X1*	rs17102977	AA	111	27	12.02	5.30 × 10^−4^/0.034 ^2^
		G allele	12	13		
*TBXAS1*	rs3819733	TT	81	35	6.76	0.009/0.130 ^2^
		C allele	46	6		

^1^ Numbers of patients with specified genotypes divided by the response to the cytotoxic neoadjuvant therapy. ^2^ Adjusted *p*-values using the false discovery rate test. ^3^ For this variant, we used the dominant genetic model; in the rest we present recessive or additive (rs593421) model; SNP = single nucleotide polymorphism.

**Table 5:** The effect of breast cancer molecular subtypes on cytochrome P450 variants significantly associating with DFS of patients in the confirmation set.

Gene	SNP ID	Genotypes	Subtypes			
			Luminal A	Luminal B	HER2	TNBC
All patients (n = 744)						
*CYP26B1*	rs62150087	CC ^1^	174	230	44	73
		G allele ^1^	36	42	12	12
		*p* ^2^	0.754	0.086	**0.010**	0.178
*CYP4X1*	rs17102977	AA ^1^	166	223	49	48
		G allele ^1^	44	42	6	16
		*p* ^2^	0.245	0.130	0.150	0.778
Patients treated with cytotoxic therapy (n = 371)						
*CYP26B1*	rs62150087	CC ^1^	65	128	26	58
		G allele ^1^	9	25	10	8
		*p* ^2^	0.244	0.232	**0.011**	0.060
*CYP24A1*	rs2762934	GG ^1^	50	91	27	45
		A allele ^1^	24	60	9	19
		*p* ^2^	0.181	0.172	0.400	**0.001**
Patients treated only with hormonal therapy (n = 311)						
*CYP4X1*	rs17102977	AA ^1^	102	81	3	1
		G allele ^1^	22	19	0	1
		*p* ^2^	0.123	0.202	N/A	0.317

^1^ Numbers of patients with genotypes/alleles; ^2^ log-rank *p*-values (significant associations are depicted in bold). HER2 = ERBB2/HER2-enriched subtype; TNBC = triple-negative breast cancer; N/A = not applicable; DFS: disease-free survival.

## Data Availability

The sequencing data that support the findings of this study are openly available in Sequence Read Archive (SRA, https://www.ncbi.nlm.nih.gov/sra), under accession No. PRJNA510917 (https://www.ncbi.nlm.nih.gov/bioproject/PRJNA510917).

## Supplementary Materials

Supplementary materials can be found at https://www.mdpi.com/1422-0067/22/6/2826/s1. Figure S1: The effect of breast cancer molecular subtypes on cytochrome P450 variants significantly associating with DFS of patients in the confirmation set, Table S1: Clinical data of patient in the testing set, Table S2: Prioritized variants for the validation phase, Table S3: Clinical data of patients in the validation set.

## Abbreviations

CI	Confidence interval
CYP	Cytochrome P450
DFS	Disease-free survival
FDR	False discovery rate
GWAS	Genome-wide association study
HR	Hazard ratio
LOF	Loss-of-function
MAF	Minor allele frequency
NACT	Neoadjuvant cytotoxic therapy
qPCR	Quantitative real-time PCR
SNP	Single nucleotide polymorphism
SNV	Single nucleotide variant
UTR	Untranslated region
