# Supplementary material for: Role of Genetic Variation in Cytochromes P450 in Breast Cancer Prognosis and Therapy Response
Source: Int J Mol Sci. 2021 Mar 10;22(6):2826. doi: 10.3390/ijms22062826 (PMC8001203; doi:10.3390/ijms22062826)

International Journal of
Molecular Sciences
MDPI

Supplementary materials

Role of Genetic Variation in Cytochromes P450 in Breast Cancer Prognosis and Therapy Response

Viktor Hlaváč [1,2]*, Radka Václavíková [1,2], Veronika Brynychová [1,2], Pavel Ostašov[2], Renata Koževnikovová [3], Kateřina Kopečková [4], David Vrána [5], Jiří Gatěk [6] and Pavel Souček [1,2]

1 Toxicogenomics Unit, National Institute of Public Health, 100 00 Prague, Czech Republic; rvaclavikova@seznam.cz (R.V.); veronikabrynychova@seznam.cz (V.B.); pavel.soucek@szu.cz (P.S.)
2 Biomedical Center, Faculty of Medicine in Pilsen, Charles University, 301 00 Pilsen, Czech Republic; pavel.ostasov@lfp.cuni.cz
3 Department of Oncosurgery, MEDICON, 140 00 Prague, Czech Republic; renata.kozevnikovova@onko-centrum.cz
4 Department of Oncology, Second Faculty of Medicine, Charles University and Motol University Hospital, 150 00 Prague, Czech Republic; katerina.kopeckova@fnmotol.cz
5 Comprehensive Cancer Center Novy Jicin, Hospital Novy Jicin, 741 01 Novy Jicin, Czech Republic; davvrana@gmail.com
6 Department of Surgery, EUC Hospital Zlin and Tomas Bata University in Zlin, 763 02 Zlin, Czech Republic; gatekj@gmail.com
* Correspondence: viktor.hlavac@szu.cz; Tel.: +420-267-082-711

International Journal of *Molecular Sciences*

MDPI

Supplementary Table S1. Clinical data of patient in the testing set.

Characteristics	Patients, N (%)[1]
Age at diagnosis, mean ± S.D.[2] (years)	51.7 ± 9.4
Menopausal status	
Premenopausal	46 (46)
Postmenopausal	55 (55)
Missing data	4
Tumor size (pT)	
pTis	8 (8)
pT1	50 (48)
pT2	40 (39)
pT3	5 (5)
pTX	2
Lymph node metastasis (pN)	
Absent (pN0)	68 (65)
Present (pN1-3)	37 (35)
Pathological stage	
SI	46 (44)
SII	47 (45)
SIII	12 (11)
Histological type	
Invasive ductal carcinoma	88 (84)
Other type	17 (16)[4]
Pathological grade	
G1	11 (11)
G2	35 (35)
G3	54 (54)
GX	5
Estrogen receptor status	
Positive	38 (38)
Negative	61 (62)
Missing data	6
Progesterone receptor status	
Positive	39 (39)
Negative	60 (61)
Missing data	6
Expression of HER2	
Positive	2 (2)
Negative	97 (97)
Missing data	6
Expression of Ki-67, mean ± S.D.[2] *(%)*	32.9 ± 20.3

International Journal of Molecular Sciences

Molecular subtype	
Luminal A	11 (11)
Luminal B	30 (30)
Triple negative	58 (59)
Missing data	6
Response to neoadjuvant cytotoxic therapy	
Complete or partial response	47 (69)
Stable disease or progression	21 (31)
Not applicable[3]	37

Footnotes:

[1] Number of patients with % in parentheses

[2] S.D.=standard deviation

[3] Patients treated with adjuvant therapy without neoadjuvant cytotoxic therapy

[4] Six lobular, six medullary, two metaplastic, one mucinous, one papillary, and one neuroendocrine invasive carcinomas

International Journal of
Molecular Sciences

MDPI

Supplementary Table S2. Prioritized variants for the validation phase.

Gene	HGVS coding (GRCh38)	HGVS protein	Classification[1]	Rs ID[2]	Response[3]	DFS[3]	MAF[4]	GnomAD[5]
CYP1B1	NC_000002.12:g.38075034C>A	NP_000095.2:p.Ala119Ser	Missense	rs1056827	0.029	NS	0.34	0.31
CYP2S1	NC_000019.10:g.41198050A>G	-	Intron	rs184623	NS	0,030	0.38	0.36
CYP2W1	NC_000007.14:g.988812C>T	-	Intron	rs3808348	0.036	NS	0.20	0.20
CYP2W1	NC_000007.14:g.983092T>C	-	Intron	rs12701220	NS	0,038	0.11	0.21
CYP4A11	NC_000001.11:g.46933071G>C	-	Intron	rs3890011	0.029	NS	0.27	0.22
CYP4F2	NC_000019.10:g.15886010G>A	NP_001073.3:p.His343=	Synonymous	rs2074900	0.006	NS	0.32	0.32
CYP4F2	NC_000019.10:g.15879238A>G	-	Intron	rs3093198	0.011	NS	0.29	0.30
CYP4F8	NC_000019.10:g.15618245C>G	-	Intron	rs714772	NS	0,011	0.25	0.20
CYP4F8	NC_000019.10:g.15615549G>T	-	Intron	rs4646522	NS	0,030	0.42	0.48
CYP4F12	NC_000019.10:g.15697020T>C	NP_076433.3:p.Leu504=	Synonymous	rs593421[6]	0.017	NS	0.29	0.27
CYP4F12	NC_000019.10:g.15697074A>G	NP_076433.3:p.Ser522Arg	Missense	rs593818	NS	0,015	0.43	0.45
CYP4F12	NC_000019.10:g.15680322T>A	-	Intron	rs2074568	0.029	NS	0.21	0.16
CYP4V2	NC_000004.12:g.186194623G>C	-	Intron	rs62350517	NS	0,007	0.08	0.06
CYP4X1	NC_000001.11:g.47029933A>G	-	Intron	rs17102977	0.013	NS	0.10	0.08
CYP24A1	NC_000020.11:g.54171775T>C	-	Intron	rs2259735	NS	0,026	0.39	0.40
CYP24A1	NC_000020.11:g.54154722G>A	-	3'-UTR	rs2762934	NS	0,018	0.17	0.19
CYP24A1	NC_000020.11:g.54171474A>G	-	Intron	rs6022999	NS	0,003	0.21	0.24
CYP24A1	NC_000020.11:g.54153515_54153516insAT	-	3'-UTR	rs10623012	NS	0,015	0.32	0.40
CYP26B1	NC_000002.12:g.72130207C>T	-	3'-UTR	rs61138718	NS	0,018	0.11	0.14
CYP26B1	NC_000002.12:g.72129135C>G	-	Downstream Transcript Variant	rs62150087	NS	0,011	0.07	0.10
CYP27C1	NC_000002.12:g.127193883C>T	-	Intron	rs12476709	0.029	NS	0.47	0.41
TBXAS1	NC_000007.14:g.139913631T>C	-	Intron	rs3819733	0.013	NS	0.15	0.14

Footnotes:

[1] Classification in Annovar

International Journal of *Molecular Sciences*

[2] SNV number in dbSNP (https://www.ncbi.nlm.nih.gov/snp/)

[3] *p*-value provided for clinical associations; NS = non-significant

[4] MAF = minor allele frequency in the evaluation set

[5] The Genome Aggregation Database (gnomAD), MAF in European non-Finnish population

[6] Variant replacing failed rs79882219 ($p = 0.011$ with response) based on tagging analysis

International Journal of *Molecular Sciences*

MDPI

Supplementary Table S3. Clinical data of patients in the validation set.

Characteristics	Patients, N (%)[1]
Age at diagnosis, mean ± S.D.[2] (years)	58.9 ± 12.5
Menopausal status	
Premenopausal	196 (25)
Postmenopausal	592 (75)
Missing data	14
Tumor size (pT)	
pTis	65 (8)
pT1	488 (62)
pT2	206 (27)
pT3	18 (2)
pT4	10 (1)
pTX	15
Lymph node metastasis (pN)	
Absent (pN0)	507 (67)
Present (pN1-3)	252 (33)
pNX	43
Pathological stage	
S0	61 (8)
SI	343 (46)
SII	282 (38)
SIII	64 (9)
SIV	1 (0)
Not determined	51
Histological type	
Invasive ductal carcinoma	596 (75)
Other type	196 (25)
Missing data	10
Pathological grade	
G1	177 (23)
G2	382 (50)
G3	209 (27)
GX	34
Estrogen receptor status	
Positive	615 (77)
Negative	181 (23)
Missing data	6
Progesterone receptor status	
Positive	577 (73)
Negative	219 (27)
Missing data	6

International Journal of
Molecular Sciences

Expression of HER2	
Positive	194 (24)
Negative	600 (76)
Missing data	8
Expression of Ki-67, mean ± S.D.[2] (%)	23.3 ± 22.6
Molecular subtype	
Luminal A	320 (40)
Luminal B	315 (40)
Triple negative	94 (12)
HER2	63 (8)
Missing data	10
Response to neoadjuvant cytotoxic therapy	
Complete or partial response	127 (76)
Stable disease or progression	41 (24)
Not applicable[3]	634

Footnotes:

[1] Number of patients with % in parentheses

[2] S.D.=standard deviation

[3] Patients treated with adjuvant therapy without neoadjuvant cytotoxic therapy

International Journal of
Molecular Sciences

MDPI

Supplementary Figure S1: The effect of breast cancer molecular subtypes on significant associations of cytochrome P450 variants with DFS of the patients in the confirmation set.

Blue line depicts for common homozygous and green for rare allele.

a) All patients (n=744)

rs6210087
HER2-enriched subtype

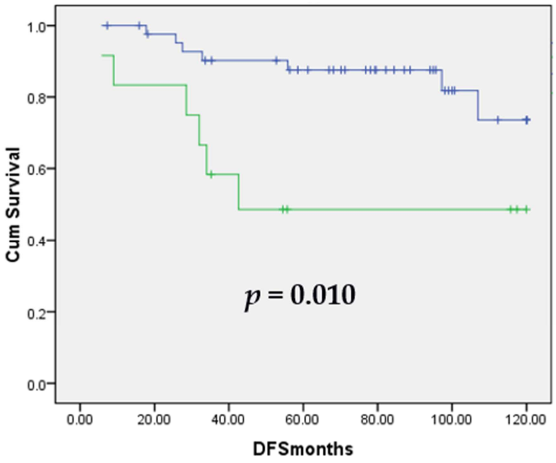

b) Patients treated with cytotoxic therapy (n=371)

rs6210087
HER2-enriched subtype

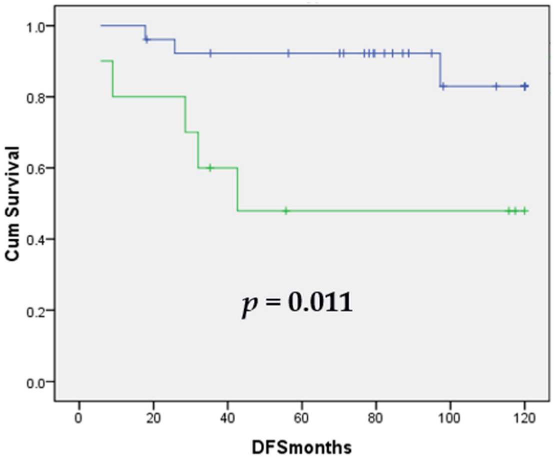

rs2762934
TNBC subtype

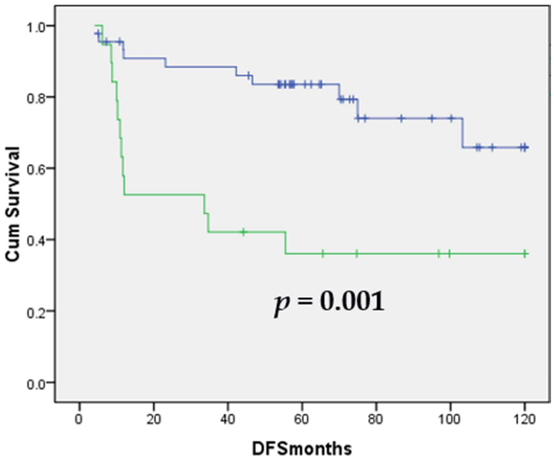

Supplement: Supplementary file 1 [file ijms-22-02826-s001.pdf]
